# Significance of Galectin-3 and N-terminal pro b-type natriuretic peptide in the prediction of atrial fibrillation after cardiac surgery

**DOI:** 10.5937/jomb0-47001

**Published:** 2024-06-15

**Authors:** Nikola Mladenović, Ranko Zdravković, Lazar Velicki, Vanja Drljević-Todić, Mirko Todić, Srđan Maletin, Aleksandra Mladenović, Nemanja Petrović, Bogdan Okiljević, Valentina Nikolić, Milan Pavlović, Dane Krtinić, Aleksandar Nikolić, Marko Gmijović, Aleksandar Kamenov

**Affiliations:** 1 Institute of Cardiovascular Diseases of Vojvodina, Sremska Kamenica; 2 University of Novi Sad, Faculty of Medicine, Novi Sad; 3 Institute of Cardiovascular Diseases Dedinje, Belgrade; 4 University of Nis, Faculty of Medicine, Nis,; 5 University of Nis, Faculty of Medicine, Nis; 6 University Clinical Center Nis, Nis

**Keywords:** Post-operative atrial fibrillation, Cardiac surgery, Galectin-3, NT-proBNP, Post-operativna atrijalna fibrilacija, kardihirurgija, Galektina-3, NT-proBNP

## Abstract

**Background:**

Post-operative atrial fibrillation (POAF) is a frequent complication after cardiac surgery. It is associated with prolonged hospital stay, increased morbidity, mortality rate and economic costs. The aim of the study was to determine the association between the values of Galectin3 and N-terminal pro-B-type natriuretic peptide (NTproBNP) with POAF after cardiac surgery.

**Methods:**

A prospective study enrolled patients aged 18-85 years old admitted due to elective coronary artery bypass graft surgery (CABG) or CABG + aortic valve replacement. The plasma Galectin-3 and NT-proBNP levels were measured one day before surgery postoperative days 1 and 7.

**Results:**

The study included a total of 103 patients. POAF was registered in 45 patients. The mean age of patients in whom POAF occurred was 68.8 years, while other patients' mean age was 65.5 years (p=0.028). Patients with POAF did not differ from the group without POAF in the values of Galectin-3 and NT-proBNP preoperatively as well as on the first and seventh postoperative days. Changes in Galectin-3 levels on the first postoperative day had statistically significant value for predicting POAF (AUC=0.627 0.509-0.745 , p<0.05). Decrease in Galectin-3 level con centration on the first postoperative day over 17% increases the risk of developing AF.

**Conclusions:**

Preoperative values of Galectin-3 and NTproBNP are not associated with POAF development after cardiac surgery.

## Abstract (sr)

**Uvod:**

Postoperativna atrijalna fibrilacije (POAF) je učestala komplikacija nakon kardiohirurških procedura. Povezana je sa produženim hospitalnim lečenjem, povećanim morbiditetom, stopom mortaliteta i finansijskim troškovima. Cilj rada je bio da se utvrdi povezanost nivoa Galektina-3 i Nterminalnog pro-B-tipa natriuretskog peptida (NT-proBNP) sa POAF nakon kardiohirurške operacije.

**Metode:**

Prospektivna studija obuhvata bolesnike starosti od 18-85 godina, hospitalizovanih radi elektivne operacije aorto koronarnog bajpasa (CABG) ili CABG sa zamenom aortnog zaliska. Nivoi Galektina-3 i NT-proBNP iz krvi mereni su preoperativno i postoperativnog 1. i 7. dana.

**Rezultati:**

U istraživanju je uključeno 103 bolesnika. PAOF je zabeležena kod 45 bolesnika. Prosečna starost bolesnika kod kojih je registrovan POAF bila je 68,8 godina, dok je kod ostalih bila 65,5 godina (n=0,028). Nivoi Galektina-3 i NT-proBNP nisu se razlikovali kod bolesnika koji su razvili POAF, a ni kod onih kojima nije registrovana POAF u sva tri merenja. Promene nivoa Galektin-3 prvog postoperativnog dana imaju statistički značajnu vrednost za predviđanje POAF (AUC=0.627 0,509-0,745 , p<0,05). Smanjenje nivoa Galektina-3 prvog postoperativnog dana za preko 17% dovodi do povećanog rizika od razvoj POAF.

**Zaključak:**

Preoperativne vrednosti Galektina-3 i NT-proBNP nisu povezani sa razvojem POAF nakon kardiohirurške intervencije.

## Introduction

Atrial fibrillation (AF) is an irregularity in the electric activity of the atria and there is a loss of contraction synchronization of the atrial chambers with cardiac chambers activity. In many cases it is associated with dilated left atrial dilatation and fibrotic myocardial changes as well. Post-operative atrial fibrillation (POAF) is one of the most common complications after cardiac surgeries. It can have paroxysmal or persistent features that may affect patients’ hemodynamic stability. POAF refers to AF that occurs in the first 30 days after a surgical intervention, lasts at least 15 minutes and requires treatment, or lasts less than 15 minutes resulting in clinical instability of a patient and requires treatment. POAF commonly develops between day 2 and day 5 after surgery [1]. It occurs in about 5% of all operated patients, but it develops more commonly in cardiac surgery patients (10–50%) [2] [3] [4].

Galectin-3 (Gal-3) belongs to lectin family and the present studies have identified it as a mediator of cardiac fibrosis due to its secretion by the macrophages, stimulating proliferation of cardiac fibroblasts, thus enabling deposition of Type I collagen in the cardiac muscle. Its role has also been registered in atherosclerosis as it stimulates monocytes accumulation and causes chronic vascular inflammation with atherosclerotic plaque destabilization. This protein is associated with fibroses, highlighting its role in extracellular matrix modulation [5].

N-terminal pro-B-type natriuretic peptide (NTproBNP) is a polypeptide composed of 23 aminoacids. Its synthesis starts with myocyte stretching when pre-pro-BNP splits into pro-BNP and enters the circulation as an active form of BNP hormone and an inactive N-terminal fragment. Thus, it is established as a marker of cardiovascular diseases. This marker has been most extensively studied in heart failure, and as a result it was also included in the European Recommendations for Heart Failure. NT-proBNP is also elevated in patients with AF, and it has been reported as a marker for prevalent AF [6].

The aim of the study was to determine the associations between the values of Gal-3 and NT-proBNP with POAF after cardiac surgeries.

## Materials and methods

This prospective, observational study enrolled patients admitted to the Clinic of Cardiovascular Surgery, Institute of Cardiovascular Diseases of Vojvodina, due to elective coronary artery bypass graft surgery (CABG), or combined surgery (CABG + aortic valve replacement) in the period from January 20^th^ to March 20^th^ 2020. The study was approved by the Ethics Committee of the Institute of Cardiovascular Diseases of Vojvodina, and each participant gave informed consent statements.

### Study population

The study included 103 patients, aged 18–85 years for which elective CABG or CABG + aortic valve replacement are indicated. Patients having any of the following criteria were excluded from the study: failure or mitral and tricuspid valve stenosis of moderate to severe degree, ejection fraction left ventricular below 35%, previous history of AF, malignancy, systemic inflammatory diseases, impaired renal function (glomerular filtration rate below 30 mL/min/1.73 m^2^), active viral infection, respiratory insufficiency, smokers, frequent use of alcoholic beverages, pregnant and breastfeeding women, refusal to participate in the study, or any other objective personal reason that prevents or complicates participation in the study. All the patients underwent standard preoperative preparation in terms of laboratory parameters tests, electrocardiograph testing (ECG), echocardiographic examination, a carotid Doppler test, abdominal ultrasound, and other necessary examinations. Inclusion in the study did not affect surgical or postsurgical treatment. All the patients underwent standard anaesthesiological and surgical procedures. Postoperative standard monitoring treatment included the Intensive, Semi-Intensive Care Units stay, and rehabilitation treatment.

### Anesthesia and surgical technique

Anesthesia was managed with combinations of drugs: sufentanil, midazolam, propofol, and rocuronium bromide. After endotracheal intubation, mechanical ventilation was performed with an oxygen/air mixture of 50:50. Anesthesia management was with assistance sevoflurane, analgesia with a continuous infusion of sufentanil, and muscle relaxation with intermittent administration of rocuronium bromide. Perioperative and postoperative monitoring included continuous arterial and central venous pressure measure ment, electrocardiography (ECG), oxygen saturation (pulse oximetry), body temperature measured in the nasopharynx, and diuresis. Monitoring of gas exchange from arterial blood was done according to the protocol and clinical condition of the patient.

All patients included in the study underwent a full midline sternotomy. After that, the left internal mammary artery was harvested extrapleuraly in a skeletonized fashion. The great saphenous vein was extracted from the lower extremities through continuous incision. With the Heparin the activated clotting time keep above 480 s. Aortic and double-stage venous cannulation were performed. The CPB (cardiopulmonary bypass) circuit was standard, with mild hypothermia (32–34°C) and ante grade intermittent cold (extracellular crystalloid or blood) cardioplegia. All anastomoses (proximal and distal) were done during a single cross-clamp period. In cases where aortic valve replacement was done in addition to CABG, aortic valve was exposed through transversal aortotomy. Aortic valve exscision, decalcification and prosthesis sizing were done before distal anastomoses. After performing distal anastomoses of CABG, aortic valve prosthesis was implanted with simple or pledgeted interrupted sutures. Aortotomy was closed with two-layer continuous suture and proximal anastomoses were created. Proper deaeration preceded cross-clamp removal. After separating the patient from extracorporeal circulation, two drains (retrocardial and retrosternal) were used with negative pressure suction. Sternal bone and wound were closed according to a standard protocol, without closure of the pericardia.

### Outcomes

The occurrence of POAF after cardiac surgery was the primary outcome. In this study, POAF was defined as any dysrhythmia that represents the ECG characteristics of atrial fibrillation lasting at least 30s on a rhythm strip or 12-lead ECG [7]. Patients from both groups were continuously monitored postoperatively in the Intensive Care Unit (ICU), as well as in the Semi-ICU later. A 12-channel ECG was performed daily in the morning, as well as after each episode of dyspnea, chest pain, and palpitations.

### Laboratory measures

Blood samples were taken from patients’ cubital vein in the preoperative period (1 day before the surgery), on postoperative day 1 and postoperative day 7. Besides performing standard preoperative and postoperative blood count, biochemical NT-proBNP and Gal-3 parameters were evaluated as well. Centrifugation and serum separation was performed in the biochemical laboratory of the Institute of Cardiovascular Diseases of Vojvodina, followed by standard preparation for deep freeze and transportation to the laboratory of the Medical Faculty in Nis where a post-thawing analysis was performed according to manufacturers’ standards for reagents »FinTest Human GAL3 ELISA Kit« and »FinTest NT-proBNP ELISA Kit« for obtaining results for Gal-3 and NT-proBNP.

This kit for both tests was based on sandwich enzyme-linked immune-sorbent assay technology. Capture antibody was precoated onto the 96-well plate. The biotin conjugated antibody was used as the detection antibody. The standards and pilot samples were added to the wells subsequently. After incubation, unbound conjugates were removed by wash buffer. Then, biotinylated detection antibody was added to bind with antigen conjugated on coated antibody. After washing off unbound conjugates, HRP-Streptavidin was added. After a third washing, TMB substrates were added to visualize HRP enzymatic reaction. TMB was catalyzed by HRP to produce a blue color product that turned yellow after adding acidic stop solution. Reading was done by spectrometric results.

### Statistical analysis

Statistical data analysis was performed by using the software package SPSS 25.0 (Statistical Package for Social Sciences; Chicago, IL, USA). The association between the concentration of analyzed biomarkers and characteristics of patients included in the study and performed interventions was tested by using parametric (Student t-test) and non-parametric tests (Mann–Whitney U-test, χ^2^-test). The correlation between continuous variables was evaluated by the Spearman correlation coefficient. The results were given as mean values with standard deviation or the median with interquartile range (depending on distribution normality) for continuous variables, or as an absolute number with frequency for qualitative variables [8] [9]. The normality of distribution was assessed according to distribution sample characteristics (asymmetry, flatness, the presence of extreme values, Shapiro-Wilk test). Independent predictors of dependent variables were identified by linear regression model, but also by binary logistic regression model. Based on the receiver operating characteristic (ROC) curve analysis, optimal cut-off values and histoscore discriminatory ability were determined. The threshold value of *p*<0.05 was considered statistically significant.

A priori, we have calculated that the sample size needed is 54, for the study power of 80% and the probability of type I error of 0.05, based on the results found in the literature (Erdem K, et al) [10]. The plasma galectin-3 level has high specificity and sensitivity for predicting postoperative atrial fibrillation after coronary artery bypass surgery). Post hoc analysis showed that the same power, but using our results as the input parameters, would be obtained with the sample of 90 patients, a number below our actual sample size.

## Results

The study included a total of 103 patients who underwent cardiac interventions. POAF was registered in 45 (44%) patients. Mean age of patients in whom POAF occurred was 68.8 years, while other patients’ mean age was 65.5 (*p*=0.028) (Table 1). The groups were not different in terms of body mass index, ejection cardiac fraction, diastolic dysfunction, size of heart cavities, type of surgeries, aortic clamp time duration and the time of extracorporeal circulation, ICU length of stay, nor for duration of hospital stay. In relation to gender (Table 2) there were no significant statistical differences as well as their body mass index. There was no statistically significant difference between the type of cardiosurgical intervention and ejection cardiac fraction, diastolic dysfunction, age, body mass index, Galectin-3 and NT-ProBNP before surgery, it was only observed that patients with combined surgical intervention were older (Table 3).

**Table 1:** Patient characteristics. BMI – Body mass index; LVIDd - Left ventricular internal diameter end diastole; CABG – Coronary artery bypass graft; ICU – Intensive Care Unit

	POAF (yes)<br>n=	POAF (no)<br>n=	p
Age, years	68.8±6.8	65.5±5.2	0.028
Male, n (%)	35 (77.8%)	53 (81.5%)	0,636
BMI, kg/m^2^	28.1±3.4	28.3±3.6	0.251
Ejection fraction (%)	52.9±9.1	49.8±10.0	0.102
Diastolic dysfunction, n (%)<br>Normal<br>Grade 1<br>Grade 2<br>Grade 3	<br>11 (26.7%)<br>23 (53.3%)<br>7 (17.8%)<br>1 (2.2%)	<br>9 (15.4%)<br>38 (60.0%)<br>13 (23.1%)<br>1 (1.5%)	0.508
Left atrial diameter, cm	3.9±0.4	4.0±0.4	0.361
LVIDd, cm	5.2 (4.7–5.5)	5.1 (4.6–5.6)	0.648
Type of surgery, n (%)<br>CABG<br>CABG + aortic valve replacement	<br>32 (71.1%)<br>13 (28.9%)	<br>43 (66.2%)<br>22 (33.9%)	0.470
EuroSCORE II	2.6 (1.4–4.1)	1.5 (0.9–2.6)	0.767
Aortic cross-clamp time, min	69.0±28.6	64.9±23.1	0.419
Cardiopulmonary bypass time, min	69.0 (59.0–114.0)	69.0 (56.0–95.0)	0.886
ICU, hours	26.0 (23.0–49.0)	27.0 (23.0–49.0)	0.801
Hospital length of stay, days	9.0 (9.0–10.0)	7.0 (7.0–9.0)	0.086

**Table 2:** Patient characteristics by gender. BMI – Body mass index

	Male	Females	t or Z or χ^2^ (p)
Age, years	66.64±8.04	68.05±7.22	0.750 (0.455)
BMI (kg/m^2^)	28.42±3.57	27.73±3.52	0.816 (0.416)

**Table 3:** Patient characteristics based on surgical intervention. CABG – Coronary artery bypass grafting, BMI – Body mass index

		CABG	CABG+ aortic valve	t or Z or χ^2^ (p)
Age, years		65.87±7.41	69.17±8.44	2.083 (0.040)
Male, n (%)		16 (21.3%)	6 (17.1%)	0.065 (0.799)
BMI (kg/m^2^)		28.52±3.66	27.77±3.34	1.028 (0.306)
Ejection fraction (%)		50.31±9.36	52.94±10.50	1.323 (0.189)
Diastolic<br>dysfunction,<br>n (%)	Normal	15 (21.3%)	5 (17.1%)	6.105 (0.107)
	Grade 1	45 (62.7%)	15 (45.7%)	
	Grade 2	10 (14.7%)	11 (34.2%)	
	Grade 3	1 (1.3%)	1 (2.9%)	
Galectin-3 (ng/mL)		0.7 (0.5–0.9)	0.8 (0.6–1.1)	1.348 (0.178)
NT-ProBNP (pg/mL)		127.5 (83.2–195.5)	160.2 (99.8–225.1)	0.532 (0.595)

Between the two groups of patients in terms of type of surgery (Table 4), there was no statistically significant difference in the measured concentration of Galectin-3 on preoperatively, 1. and 7. day after surgical intervention. However, on 1. day in patients after the combined intervention, there was a greater decrease in the concentration of Galectin-3 (*p*<0.05). Similarly, compared to basal values, a greater drop in concentration in the first week after the intervention occurred in patients after the combined intervention (*p*<0.05).

**Table 4:** Type of surgical intervention and Galectin-3. CABG – Coronary artery bypass grafting

	CABG	CABG+ aortic valve replacement	Z (p) or χ^2^ (p)
0. day (ng/mL)	0.7 (0.5–0.9)	0.8 (0.6–1.1)	1.348 (0.178)
1. day (ng/mL)	0.7 (0.5–0.9)	0.6 (0.5–0.7)	1.145 (0.252)
7. day (ng/mL)	0.4 (0.4–0.6)	0.4 (0.3–0.5)	1.227 (0.220)
0. day >10 ng/mL	1 (1.5%)	0 (0.0%)	0.000 (1.000)
1. day >10 ng/mL	1 (1.5%)	0 (0.0%)	0.000 (1.000)
7. day >10 ng/mL	0 (0.0%)	0 (0.0%)	0.000 (1.000)
1–0 (ng/mL)	0.0 (-0.3–0.1)	-0.2 (-0.6–0.0)	2.246 (0.025)
7–1 (ng/mL)	-0.2 (-0.4–0.1)	-0.2 (-0.3–0.1)	0.780 (0.435)
7–0 (ng/mL)	-0.2 (-0.5–0.1)	-0.4 (-06–0.2)	2.020 (0.043)
1/0 (ng/mL)	1.0 (0.6–1.3)	0.7 (0.6–1.0)	2.220 (0.026)
7/1 (ng/mL)	0.6 (0.5–0.8)	0.7 (0.6–0.8)	0.772 (0.440)
7/0 (ng/mL)	0.6 (0.4–0.9)	0.5 (0.3–0.7)	1.986 (0.047)

Between the two groups of patients in terms of type of surgery (Table 5), there was no statistically significant difference in the measured concentration of NT-ProBNP on preoperatively, 1. and 7. day after surgical intervention.

**Table 5:** Type of surgical intervention and NT-ProBNP. CABG – Coronary artery bypass grafting

	CABG	CABG+ aortic valve replacement	Z (p) or χ2 (p)
0. day (pg/mL)	127.5 (83.2–195.5)	160,2 (99.8–225.1)	1.132 (0.258)
1. day (pg/mL)	111.8 (74.8–178.8)	105,9 (85.7–190.6)	0.208 (0.835)
7. day (pg/mL)	92.1 (56.6–159.1)	100,0 (56.2–242.8)	0.429 (0.668)
0. day >125 pg/mL ili 450 pg/mL	32 (48.5%)	11 (42.3%)	0.092 (0.640)
1. day >125 pg/mL ili 450 pg/mL	27 (40.9%)	9 (34.6%)	0.102 (0.641)
7. day >125 pg/mL ili 450 pg/mL	23 (34.8%)	9 (34.6%)	0.000 (1.000)
1–0 (pg/mL)	-31.0 (-87.7–55.2)	-33.3 (-89.7–4.9)	0.676 (0.499)
7–1 (pg/mL)	-17.7 (-72.9–32.5)	-19.5 (-40.2–77.8)	0.724 (0.469)
7–0 (pg/mL)	-31.5 (-85.7–41.6)	-43.3 (-127.1–71.9)	0.139 (0.890)
1/0	0.7 (0.5–1.7)	0.7 (0.5–1.0)	0.529 (0.597)
7/1	0.8 (0.5–1.3)	0.8 (0.6–1.6)	0.520 (0.603)
7/0	0.7 (0.4–1.4)	0.6 (0.3–1.5)	0.208 (0.835)

**Figure 1:**
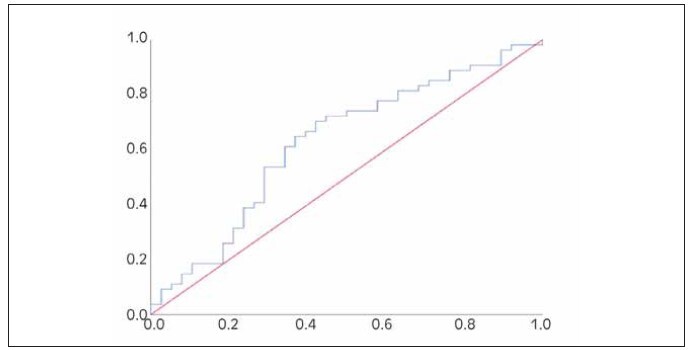
Comparing serum biomarker concentrations between the LC group and the benign group, (A) serum CEA levels, (B) serum SCC levels, (C) serum CYFRA21-1 levels. *P < 0.05.

Patient in groups with and without POAF did not show any differences in preoperative values of Gal-3 nor in the values registered on postoperative day 1. and day 7. (Table 6). Changes in Gal-3 levels on the first postoperative day have statistically significant value for predicting POAF (AUC=0.627 [0.509–0.745], *p*<0.05) (Figure 1). Optimal cut-off value is 0.83 ng/ml (sensitivity 70.4%, specificity 57.9%). Patients experiencing a decrease in Gal-3 level concentration on the first postoperative day for over 17% are at an increased risk of developing AF.

**Table 6:** Study population plasma Galectin-3 level. POAF – Post-operative atrial fibrillation

Galectin-3 ng/mL	POAF (yes)	POAF (no)	p
The day before the operation	0.8 (0.5–1.2)	0.7 (0.5–0.9)	0.390
The first postoperative day	0.6 (0.5–0.8)	0.6 (0.5–0.9)	0.341
The seventh postoperative day	0.4 (0.3–0.5)	0.4 (0.4–0.6)	0.339

Preoperative values of NT-proBNP were not statistically significantly different in patients who developed POAF in comparison to other patients (Table 7). Also, the values were not different on postoperative day 1. and day 7.

**Table 7:** Study population NT-proBNP level. POAF – Post-operative atrial fibrillation

NT-proBNP pg/mL	POAF (yes)	POAF (no)	p
The day before the operation	127.5 (82.2–186.7)	139.4 (100.0–214.2)	0.216
The first postoperative day	100.0 (71.8–143.3)	125.6 (83.2–186.7)	0.237
The seventh postoperative day	76.3 (52.7–142.3)	113.8 (60.6–161.0)	0.185

## Discussion

POAF is the most common heart rhythm disorder after cardiac surgeries and is associated with prolonged hospital stay, increased morbidity and mortality rate, and generates increased economic costs [4] [8] [9]. Preexisting atrial fibrotic remodeling, oxidative stress, active pulmonary vein sleeves, advanced age, inflammation, and pressure and volume overload are possible underlying mechanisms for the development of POAF following CABG surgery [10].

Investigations of Gal-3 and NT-proBNP have been conducted in a variety of cardiac diseases, such as heart failure, myocardial fibrosis, atrial fibrosis, myocardial infarction, ischemic cardiomyopathy [11] [12] [13]. In this study that included 103 adult patients of both genders in whom CABG surgery, or CABG combined with aortic valve replacement was performed, Gal-3 and NT-proBNP were monitored in the preoperative, early postoperative and late postoperative period in relation to POAF development. The results of our study did not find the correlation between preoperative values of these two biomarkers and POAF occurrence. What is particularly interesting in our study is the fact that Gal-3 level changes in postoperative day 1 have statistically significant value in predicting POAF development. Patients in whom Gal-3 values decrease for more than 17% are at an increased risk of POAF development.

A study by Richter et al. [14] assessed the association between Gal-3 values and POAF development after CABG surgery [10]. They showed that this profibrotic marker is an independent predictor of POAF and mortality. The study included 475 patients and Gal-3 levels were assessed on the same day after cardiac surgery. A study measuring the association between Soluble suppression of tumorigenicity 2 (ST2) and Gal-3 with cardiovascular events and mortality after cardiac surgery with more than 1800 patients included showed the association between higher perioperative values of these two biomarkers and adverse cardiovascular events and mortality [15]. Unlike our study, a similar study by Polineni et al. [16] demonstrated that preoperative Gal-3 levels and NT-proBNP levels were highly associated with intra-hospital mortality after CABG surgery. The results different from ours were found in a study by Erdem et al who investigated Gal-3 as a highly sensitive marker of POAF after CABG surgery [10].

Since the prevalence of AF has increased, assessments of Gal-3 levels were also performed in patients having no cardiac interventions. A meta-analysis including 28 studies and more than 10,000 patients showed that Gal-3 level was higher in patients with persistent AF in comparison to paroxysmal AF, with Gal-3 level generally elevated in both groups, and it can predict AF development and recurrence after treatment as well [17].

So far there have not been many studies to assess the association of NT-pro BNP with POAF occurrence after cardiac surgery. Our study did not find the association between preoperative NT-proBNP value and POAF development, unlike a similar study conducted by Xu et al. [18] who found the association between increased values of NT-proBNP preoperatively and POAF. Furthermore, a group of authors assessing this association on 215 patients reported close correlation of high pre- and post-operative values with POAF development in patients after CABG surgery [19].

Based on our study and recent studies we considered, it can be seen that further ones are needed to form an adequate database for these two biomarkers potential utility in clinical practice regarding POAF prediction and its risk levels, since POAF is one of the most common complications following cardiac surgeries.

### Limitations of the study

The study has its limitations. It is a single-center study. The sample size is relatively small, which may undermine the power of the study. Left atrium (LA) volume index was not observed, so LA diameter was used as a marker.

### Conclusion

The conclusion of our study is that preoperative values of Gal-3 and NT-proBNP biomarkers are not associated with POAF development after cardiac surgeries.

## Dodatak

### Conflict of interest statement

All the authors declare that they have no conflict of interest in this work.

### List of abbreviations

Post-operative atrial fibrillation (POAF),<br>atrial fibrillation (AF)<br>N-terminal pro-B-type natriuretic peptide (NT-proBNP)<br>Galectin-3 (Gal-3) <br>coronary artery bypass graft surgery (CABG)<br>electrocardiograph testing (ECG)<br>intensive care unit (ICU)<br>receiver operating characteristic (ROC)<br>Soluble suppression of tumorigenicity 2 (ST2)<br>left atrium (LA)
